# Dimerized Translationally Controlled Tumor Protein-Binding Peptide Ameliorates Atopic Dermatitis in NC/Nga Mice

**DOI:** 10.3390/ijms18020256

**Published:** 2017-01-26

**Authors:** Xing-Hai Jin, Juhyeon Lim, Dong Hae Shin, Jeehye Maeng, Kyunglim Lee

**Affiliations:** Graduate School of Pharmaceutical Sciences, College of Pharmacy, Ewha Womans University, Seoul 120-750, Korea; jxh630@gamil.com (X.-H.J.); rthyeon@nate.com (J.L.); dhshin55@ewha.ac.kr (D.H.S.)

**Keywords:** atopic dermatitis, dimerized TCTP, dTBP2, histamine releasing factor

## Abstract

Our previous study showed that dimerized translationally controlled tumor protein (dTCTP) plays a role in the pathogenesis of allergic diseases, such as asthma and allergic rhinitis. A 7-mer peptide, called dTCTP-binding peptide 2 (dTBP2), binds to dTCTP and inhibits its cytokine-like effects. We therefore examined the protective effects of dTBP2 in house dust mite-induced atopic dermatitis (AD)-like skin lesions in Nishiki-nezumi Cinnamon/Nagoya (NC/Nga) mice. We found that topical administration of dTBP2 significantly reduced the AD-like skin lesions formation and mast cell infiltration in NC/Nga mice, similarly to the response seen in the Protopic (tacrolimus)-treated group. Treatment with dTBP2 also decreased the serum levels of IgE and reduced IL-17A content in skin lesions and inhibited the expression of mRNAs of interleukin IL-4, IL-5, IL-6, IL-13, macrophage-derived chemokine (MDC), thymus and activation-regulated chemokine (TARC) and thymic stromal lymphopoietin (TSLP). These findings indicate that dTBP2 not only inhibits the release of Th2 cytokine but also suppresses the production of proinflammatory cytokines in AD-like skin lesions in NC/Nga mice, by inhibiting TCTP dimer, in allergic responses. Therefore, dTCTP is a therapeutic target for AD and dTBP2 appears to have a potential role in the treatment of AD.

## 1. Introduction

Atopic dermatitis (AD) is a common chronic inflammatory skin disease characterized by pruritic and eczematous lesions. AD, which affects 10%–20% of people worldwide, is rapidly increasing in developed countries [1,2]. AD is associated with food allergy, asthma, and allergic rhinitis. Glucocorticoids and immunosuppressive agents are widely used as treatment modalities for AD [3,4]. However, since prolonged use of high-dose glucocorticoids or topical calcineurin inhibitors such as tacrolimus is associated with various side-effects [5], it is imperative to develop new therapeutic agents for AD. 

Translationally controlled tumor protein (TCTP), also called histamine releasing factor (HRF), p21, p23, and fortilin, is ubiquitously expressed in various tissues, cells and even in several parasites [6,7,8,9,10]. TCTP, a house-keeping protein, is involved in a variety of cellular activities, including microtubule stabilization, calcium-binding activities, apoptosis and allergy [11,12]. It has been shown previously that TCTP appears in some cell line culture fluids and in sera of patients with asthma and atopic patients as well as in bronchoalveolar lavage fluids (BALF) of mice with airway inflammation [12] and that TCTP promotes the release of histamine from patients with atopic dermatitis and food allergies [13]. Our previous study showed that dimerized TCTP (dTCTP) is found in allergic fluids from mice with airway inflammation and from patients with atopic dermatitis. Moreover, dTCTP, not its monomeric form, is involved in the regulation of allergic reactions [12]. Dimerization of extracellular TCTP can exert cytokine-like activities whereas monomer TCTP is involved in the majority of intracellular functions of TCTP [12].

A 7-mer peptide, called dTCTP-binding protein 2 (dTBP2), binds to dTCTP and inhibits its cytokine-like effects [14,15]. We previously demonstrated that dTBP2 ameliorated ovalbumin (OVA)-induced allergic rhinitis symptoms in BALB/c mice, and reduced IL-8 release in human bronchial epithelial cells (BEAS-2B) [15]. Because dTBP2 is a potential therapeutic agent of allergic diseases by targeting dTCTP, we evaluated its potential therapeutic effect on atopic dermatitis in house dust mite-induced AD-like dermatitis in Nishiki-nezumi Cinnamon/Nagoya (NC/Nga) mice.

## 2. Results

### 2.1. Effect of dTBP2 on House Dust Mite-Induced AD-Like Skin Lesions in NC/Nga Mice

**Figure 1:**
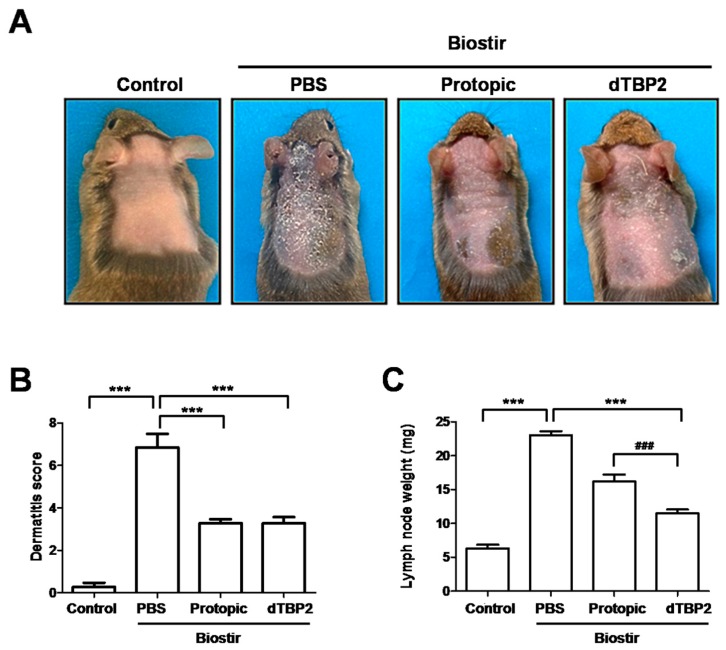

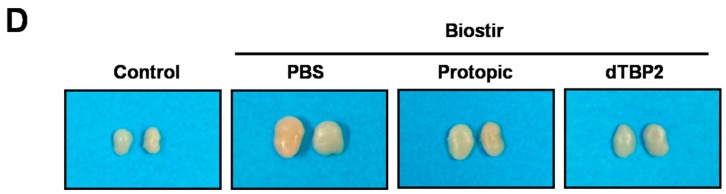
Dimerized translationally controlled tumor protein-binding peptide 2 (dTBP2) ameliorates house dust mite-induced atopic dermatitis (AD)-like skin lesions and lymph node in Nishiki-nezumi Cinnamon/Nagoya (NC/Nga) mice. Biostir AD (100 mg) was applied twice every week for 3 weeks. Protopic (50 mg) was topically applied to the same area on mice 4 times per week for 3 weeks. PBS or dTBP2 (25 mg/kg) were injected subcutaneously 4 times per week. (**A**) Typical photographs of clinical features of skin after treatment for 3 weeks in NC/Nga mice (*n* = 5); (**B**) The severity of dermatitis was determined on experimental days 1, 8, 15, and 22 from the sum of all individual scores. Atopic dermatitis scores are expressed as the mean ± SEM (*n* = 5); (**C**) Following the 3 weeks of experiments, the axillary lymph node was excised from NC/Nga mice and the weights of axillary lymph node are expressed as the mean ± SEM (*n* = 5); and (**D**) the representative photographs were presented. *** *p* < 0.0001, treatment group compared with Biostir-PBS group; ### *p* < 0.0001, dTBP2-treated group compared with Protopic-treated group.

We investigated the effect of dTBP2 on the house dust mite-induced AD-like skin lesions in this mouse model. Typical photographs of clinical features in NC/Nga mice after removing the hair from the dorsal surface of the mouse are shown in Figure 1A. After repeated applications of Biostir AD ointment, the skin became thick, and showed signs of severe erythema, hemorrhage, edema, scarring, erosion, and excoriation. Application of dTBP2 inhibited the formation of these AD-like skin lesions (Figure 1A). The clinical severity score for skin lesions for each of the four symptoms were evaluated and the sum of the individual scores was taken as the dermatitis score at 3 weeks (Figure 1B). It can be seen that the dermatitis score was significantly lower in the dTBP2-treated group, compared to PBS-treated AD mice (*p* < 0.001) and that this effect is comparable to that of immune depressant tacrolimus (Protopic)-treated AD mice.

We also evaluated how dTBP2 affects the weights of the immune organs by inhibiting the immune response by weighing the axillary lymph nodes (Figure 1C,D). After repeated applications of Biostir AD ointment, the axillary lymph node weight was increased compared with that of the control group. Application of dTBP2 significantly reduced axillary lymph node weights compared with the Biostir-PBS group (*p* < 0.001). These results indicate that dTBP2 suppresses AD-like skin lesions and reduces axillary lymph node weights, possibly through the inhibition of the leukocyte recruitment caused by the house dust-mite induced AD in NC/Nga mice.

### 2.2. Effect of dTBP2 on Tissue Inflammation and Mast Cell Infiltration in AD-Like Skin Lesions

**Figure 2:**
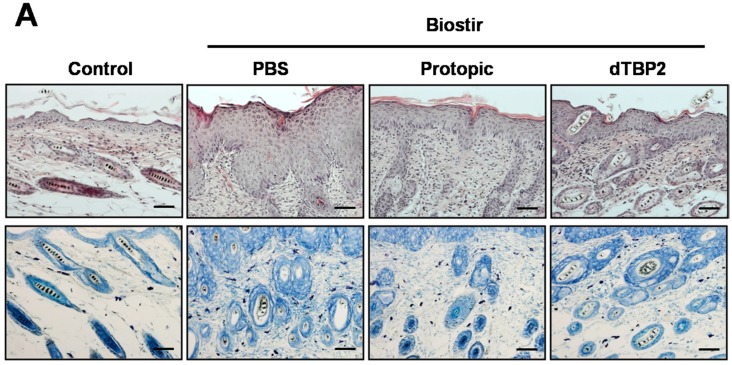

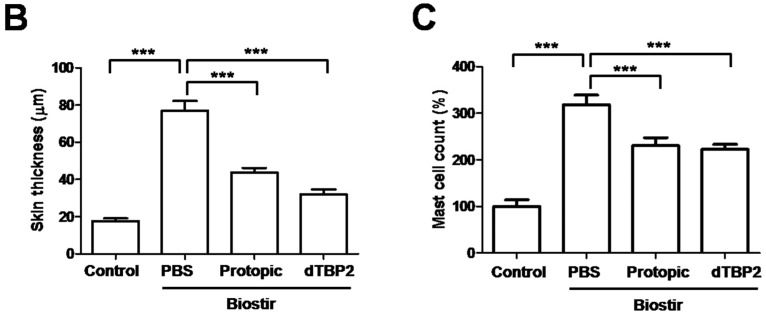
dTBP2 suppresses tissue inflammation and mast cell accumulation in AD-like skin lesions. (**A**) After sacrifice, tissue samples were excised from the back skin of each mouse. Tissue sections from NC/Nga mice were stained with hematoxylin and eosin (H&E) or toluidine blue and its histological features were examined under the microscope. Scale bar = 50 μm; (**B**) Epidermis thicknesses are assessed and expressed as the mean ± SEM (*n* = 5 sections each); (**C**) The number of mast cells was counted from five randomly selected low-power fields (*n* = 5 sections each). *** *p* < 0.0001, treatment group compared with Biostir-PBS group.

We next examined the effect of dTBP2 on the thickness of epidermis and the mast cell infiltration in the skin tissues. Figure 2A shows the representative results of the histological features of the skin lesions. Hyperplasia and thickening of the epidermis was evident and cell infiltration was more marked in PBS-treated, Biostir-induced AD mice. Application of dTBP2 inhibited tissue swelling and mast cell infiltration. The epidermal thickness was reduced by 56.7% in the dTBP2-treated group compared with the Biostir-treated group (*p* < 0.001) (Figure 2B). Skin thickness amelioration was more prominent in the dTBP-treated group than in the Protopic-treated group. Mast cell infiltration was also reduced by 27.6% in the dTBP2-treated group compared to the Biostir group (*p* < 0.001) (Figure 2C). These results indicate that dTBP2 suppresses the hyperplasia of the epidermis, as well as the infiltration of mast cells into the skin lesions of NC/Nga mice.

### 2.3. Effect of dTBP2 on IgE, Histamine and Cytokines in the House Dust Mite-Induced AD Mouse Model

**Figure 3:**
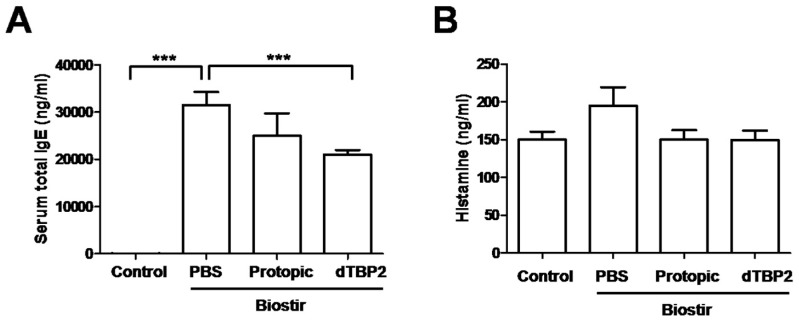

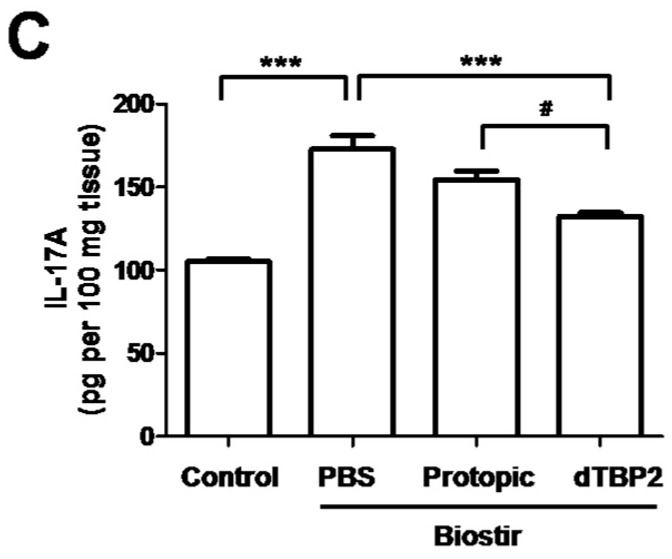
dTBP2 reduces the levels of IgE, histamine, and IL-17A in the house dust mite-induced AD mouse model. (**A**,**B**) Blood samples were collected immediately by making a puncture in the heart after sacrifice. Cells were removed by centrifugation and the plasma was carefully transferred into a new tube. The serum levels of IgE and histamine were measured using specific ELISA kits according to the manufacturer’s instructions (*n* = 5); (**C**) After sacrifice, dorsal skin biopsies were taken from each group, homogenized, and analyzed for the levels of IL-17A using ELISA kits (*n* = 5). Data are presented as the mean ± SEM of one experiment performed in triplicate. *** *p* < 0.0001, treatment group compared with Biostir-PBS group; # *p* < 0.05, dTBP2-treated group compared with Protopic-treated group.

Serum IgE and Th2 cytokine levels correlate well with the clinical severities of AD in NC/Nga mice [16,17]. We investigated whether dTBP2 also regulates the levels of IgE and Th2 cytokines in the house dust mite-induced AD mouse model. The serum IgE level increased in the PBS-treated, Biostir–induced AD group (Figure 3A). In the dTBP2-treated AD mice, the serum IgE levels were much lower compared with the PBS-treated AD mice (*p* < 0.001). The effect of dTBP2 on IgE reduction also appears to be more marked than in Protopic-treated AD mice. Histamine is regarded as one of the prime mediators of itching in AD, and is present at high levels in house dust mite-induced AD mouse model. The serum histamine levels in the PBS-treated AD group were elevated compared to the control group (Figure 3B). Application of dTBP2 tends to suppress histamine levels in the serum compared to the PBS-treated AD group. However, there was no significant difference between the dTBP2-treated AD group and the PBS-treated AD group. IL-17A has a critical role in the pathogenesis of AD and is present at high levels in AD skin lesions. The level of IL-17A greatly increased in AD-like skin lesions of PBS-treated AD mice, and was lower in dTBP2-treated or Protopic-treated group (Figure 3C). These results indicate that dTBP2 reduces IgE and histamine in serum and IL-17A in AD-like skin lesions, thereby contributing to the inhibition of the development of AD lesions in vivo.

**Figure 4:**
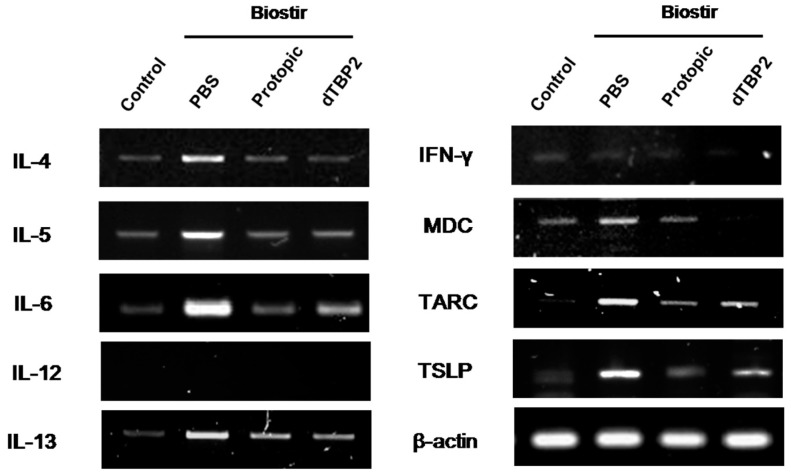
dTBP2 inhibits the cytokines/chemokines related to the pathogenesis of atopic dermatitis in house dust mite-induced AD mouse model. Total RNA was isolated from dorsal skin using a Trizol reagent kit according to the manufacturer’s instructions. Cytokine mRNA was analyzed by RT-PCR using specific primers for IL-4, IL-5, IL-6, IL-12, IL-13, IFN-**γ**, MDC, TARC, TSLP, and β-actin. PCR products were resolved on a 1.5% agarose gel and visualized with ultraviolet light after SYBR green-safe staining. This experiment was performed in triplicate.

We next examined the effect of dTBP2 on cytokine mRNA expression in skin lesions in our AD mouse model. As shown in Figure 4, mRNA expression levels of Th2 and proinflammatory cytokines increased after treatment with Biostir AD ointment in NC/Nga mice. Application of dTBP2 inhibited the mRNAs levels of proinflammatory cytokines such as IL-6 well as Th2-related cytokines/chemokines associated with pathogenesis of atopic dermatitis including IL-4, IL-5, IL-13, TSLP, MCD, and TARC (Figure 4). However, mRNA levels of IL-12 and IFN-γ were not significantly affected (data not shown). These results indicate that dTBP2 inhibits the AD symptoms through the inhibition of mRNA expression of Th2 and inflammation cytokines in AD-like skin lesions.

### 2.4. Effect of dTBP2 on the Expression of dTCTP in AD-Like Skin Lesions

**Figure 5:**
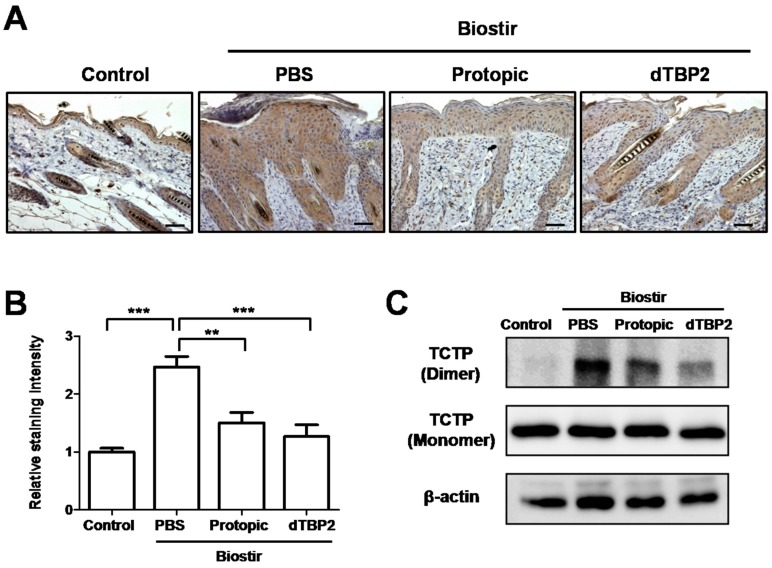
dTBP2 suppresses level of dTCTP in AD-like skin lesions. (**A**) Dorsal skin sections were stained by immunohistochemistry using mouse monoclonal anti-TCTP antibody. Immunolocalization of TCTP in AD-like skin lesions was examined under microscope. Scale bar = 50 μm; (**B**) The relative staining intensity was calculated using ImageJ software. The relative staining intensities are expressed as the mean ± SEM (*n* = 5 sections each). ** *p* < 0.001, *** *p* < 0.0001, treatment group compared with Biostir-PBS group; (**C**) Total protein was isolated from dorsal skin tissues from experimental mice and the expression of TCTP was analyzed by western blot using anti-TCTP antibody.

Dimerized TCTP is found in the bronchoalveolar lavage fluids (BALFs) from mice with airway inflammation and in serum of atopic patients [12]. We investigated whether dTBP2 induces changes of dTCTP levels in the AD-like skin lesions. Figure 5A shows the immunolocalization of TCTP in AD-like skin lesions using anti-TCTP antibody. In AD-like skin lesions from PBS-treated, Biostir-induced AD mice, strong TCTP deposition was found in epidermis and hair follicles, and moderate TCTP deposition was found in dermis. In the dTBP2-treated group, TCTP deposition was relatively weaker than that of PBS-treated AD mice, and was similar to that of the Protopic-treated AD mice. However, very little TCTP deposition was observed in the control mice. Figure 5B shows the relative staining intensities as determined by ImageJ software analysis. Because dimerization of TCTP is necessary for its cytokine-like activity and ability to cause allergic diseases [12], we investigated whether elevated level of TCTP is associated with the dimer form of TCTP. Skin lysates were prepared and subjected to western blot analysis. As shown in Figure 5C, the expressions of dTCTP increased after treatment with Biostir-AD ointment, and were reduced by treatment with TBP2 or Protopic. These results indicate that dTBP2 causes a reduction of dTCTP level in AD-like skin lesions in vivo.

## 3. Discussion

Our previous study showed that TCTP was found in the culture supernatants of several cell lines, in sera from atopic/asthmatic patients, and in BALFs from mice with airway inflammation [12]. In this study, we investigated the expression and immunolocalization of TCTP in house dust mite-induced AD-like skin lesions. Strong TCTP deposition was found in epidermis and hair follicles in AD-like skin lesions (Figure 5A) with an increase in its dimerized form (Figure 5C), whereas negligible expression of dimerized TCTP was found in the skins from normal control mice. Deposition of TCTP in skin lesions decreased after dTBP2 or Protopic treatment (Figure 5A) and the decreased form of TCTP was the dimer rather than the monomer (Figure 5C). These results indicate that TCTP is functionally involved in the pathogenesis of atopic dermatitis, and, that specifically, the dimerized TCTP was responsible for its cytokine-like activity. Dimerized TCTP is a potential pharmaceutical target for the treatment of atopic dermatitis [12]. This suggestion is supported by the observation that reduction of symptoms and AD-like lesions by dTBP2 in vivo is associated with the decrease of dTCTP levels.

A 7-mer peptide, named dTBP2, binds to dTCTP, and inhibits the cytokine-like effects of dTCTP possibly through the perturbation of dTCTP binding to dTCTP-specific receptor. Our previous study established the efficacy of dTBP2 in alleviating allergic inflammation such asthma and rhinitis [15] in mice. Here, we tested whether dTBP2 is also effective for AD treatment using NC/Nga mice, which have been used extensively for evaluating drug candidates for treatment of AD. Our results showed that repeated topical application of Biostir AD ointment resulted in AD-like skin lesions in NC/Nga mice and that application of dTBP2 markedly improved AD-like skin lesions. The severities of AD-like skin lesions were evaluated by dermatitis score including the four symptoms of AD (erythema/hemorrhage, scaling/dryness, edema and excoriation/erosion) [18] and by the lymph node assay, a common method to evaluate the proliferative responses of the local lymph node [19]. dTBP2 significantly reduced the dermatitis score and axillary lymph node weight (Figure 1). Histological analysis showed hyperplasia and thickening of the epidermis and more marked cell infiltration in Biostir-induced AD mice. In contrast, the application of dTBP2 inhibited tissue thickening and mast cell infiltration (Figure 2). These results collectively suggest that dTBP2 suppresses the Biostir AD ointment-induced spontaneous dermatitis in NC/Nga mice through the inhibition of dTCTP and its cytokine-like effects.

T helper (Th) cell differentiation is well known to be influenced during the pathogenesis process of AD. The imbalance of Th2/Th1 cell cytokines is the critical characteristic of this process [20,21]. In the acute phase of AD, activation of Th2 cells elevates the levels of Th2 cell cytokines such as IL-4, IL-5 and IL-13. In chronic phase of AD, activation of Th1 cells increases the levels of Th1 cytokines such as IFN-γ, IL-12 and GM-CSF [22]. Serum IgE and Th2 cytokine levels correlate well with the clinical severities of AD in NC/Nga mice [16,17]. In this study, total serum IgE levels were remarkably increased by repeated application of Biostir AD ointment in NC/Nga mice. Application of dTBP2 in AD mice results in the downregulation of total serum IgE levels (Figure 3A). Kang et al. [23] characterized HRF as a B cell growth factor because it increased immunoglobulin (Ig) production from B cells. It bound to B cells and in vivo treatment of HRF increased the synthesis of Igs, indicating that HRF has a role in the stimulation of B cell activation and function. In addition, Kashiwakura et al. reported that HRF interacts with specific subset of IgE and IgG antibodies [24]. Taken together, the possible interaction of dTCTP with IgE on B cells and how dTBP2 inhibits the IgE synthesis are to be verified in our future research. 

Histamine is regarded as the mediator of itching in AD, and is present at high levels in a house dust mite-induced AD mouse model. A previous study showed that TCTP was responsible for spontaneous release of histamine in patients with atopic dermatitis and food hypersensitivity [13]. Our results show that application of dTBP2 suppresses Biostir AD ointment-induced serum histamine elevation (Figure 3B). IL-17A is known to play a critical role in the pathogenesis of AD and is present at high levels in AD skin lesions [25]. The level of IL-17A in skin lesions s greatly increased in the Biostir-induced AD group, and was reduced in dTBP2- or Protopic-treated AD mice (Figure 3C). These results suggest that dTBP2 suppresses the Biostir AD ointment-induced total IgE, proinflammatory cytokine IL-17A, and histamine in NC/Nga mice. 

Langerhans cells in skin tissues produces inflammatory cytokines such as IL-1β, IL-8, GM-CSF and tumor necrosis factor-α (TNF-α) in AD skin lesions [26]. Our previous study showed that IL-4 production was significantly increased by dimerized TCTP (dTCTP) treatment in CD4^+^ Th cells [12]. A subsequent study showed that dTBP2 reduced IL-8 release by dTCTP treatment and inhibited several allergic symptoms in the animal model of allergic rhinitis [15]. Application of dTBP2 inhibited the mRNA expression of Th2 cytokines such as IL-4, IL-5, and IL-13, and epithelial cell cytokine, thymic stromal lymphopoietin (TSLP). Also, the mRNA levels of TSLP-regulated Th2-type chemokines including macrophage-derived chemokine (MDC) and thymus and activation-regulated chemokine (TARC) as well as proinflammatory cytokines such as IL-6 were found to be decreased in the skin lesions from dTBP2-treated AD mice (Figure 4). Further studies using the qRT-PCR are needed to quantitate the dTBP2-induced cytokine regulations. These results indicate that dTBP2 not only inhibited the release of Th2 cytokines but also suppressed the production of Th2 cytokines and proinflammatory cytokines. It appears that dTBP2 inhibits the AD-like symptoms through mechanisms similar to those of Protopic (tacrolimus, a calcineurin inhibitor), but mechanisms of dTBP2-induced amelioration of AD need to be further clarified.

In conclusion, dTCTP is involved in the pathogenesis of atopic dermatitis. This is supported by the finding that dTBP2 inhibits the production of Th2 cytokines in house dust mite-induced AD mice, and suppresses the production of inflammatory cytokines in vivo. The present study suggests that dimerized TCTP and dTBP2 are potential candidate drug targets and drugs in the therapy of AD.

## 4. Materials and Methods

### 4.1. Preparation of dTBP2

Synthetic dTBP2, purified to over 98% purity was provided by A & PEP Inc., Yeongi, Korea. We confirmed its purity and identity employing various criteria (high-performance liquid chromatography, mass spectroscopy).

### 4.2. Experimental Animals

All experimental procedures involving animals were conducted in accordance with the recommendations in the Guide for the Care and Use of Laboratory Animals of the National Institutes of Health and was approved by the Institutional Animal Care and Use Committee (IACUC) of Ewha Womans University (Permit Number: 15-032, Approval date: 6 June 2015). All efforts were made to minimize suffering of animals and all invasive procedures were performed under anesthesia using Zoletil^®^-Rompun^®^ mixture. Female NC/Nga mice (5 weeks old) were purchased from Charles River Laboratories (Charles River, Seoul, Korea). The animals were housed under a pathogen-free condition and maintained at controlled temperature (22 ± 2 °C) and humidity (50% ± 10%). Standard diet (Cargill Agri Purina, Seongnam, Korea) and water were supplied ad libitum. 

### 4.3. Induction of Allergic Dermatitis and Treatment in NC/Nga Mice

Biostir-AD^®^ ointment that contains an extract from house dust mite (*Dermatophagoides farinae*) was purchased from Biostir Inc. (Biostir Inc., Kobe, Japan). Mice were divided into four groups (Control group, Biostir AD ointment plus PBS group, Biostir AD ointment plus Protopic^®^ ointment group, and Biostir AD ointment plus dTBP2 group; *n* = 7/group). AD-like skin lesions were induced in 8-week-old NC/Nga mice using Biostir AD ointment. Briefly, the hair on the upper back was shaved off with an electric clipper, and depilatory cream was applied to remove the residual hairs. 200 µL of 4% (*w*/*v*) sodium dodecyl sulfate was applied on the shaved dorsal skin for barrier disruption. After 3 h, 100 mg of Biostir AD ointment was applied twice every week for 3 weeks. Protopic ointment (50 mg) was topically applied to the same area on mice 4 times per week for 3 weeks. PBS or dTBP2 (25 mg/kg) were injected subcutaneously 4 times per week. Mice were sacrificed on the last day of dTBP2 treatment. Blood was collected from mice hearts, and lymph nodes were excised for histological analysis at the time of sacrifice. The dorsal skin was excised and divided into two specimens, one for mRNA and protein extraction and the other for histological analysis.

### 4.4. Western Blot

The dorsal skins were homogenized in a lysis buffer containing 50 mM Tris-HCl (pH 7.4), 150 mM NaCl, 1 mM EDTA, 2 mM Na_3_VO_4_, 1 mM NaF, 0.25% deoxycholate, 1% Triton-X, and protease inhibitor cocktail (Roche, Mannheim, Germany). The tissue suspension was rotated at 4 °C for 10 min. Lysates were then centrifuged for 10 min at 12,000× *g* at 4 °C, and the supernatants were collected, kept at −70 °C, and used for western blotting. The proteins (25 µg) from the tissue were fractionated by non-reducing sodium dodecyl sulfate polyacrylamide gel electrophoresis (SDS-PAGE) and transferred to nitrocellulose membrane (Bio-Rad, Hercules, CA, USA). The membrane was blocked using 3% bovine serum albumin (USB, Cleveland, OH, USA) in TBS-T buffer containing 20 mM Tris (pH 7.6), 130 mM NaCl, and 0.1% Tween 20 solution for 1 h. The membrane was incubated with rabbit monoclonal anti-TCTP (Novus Biologicals, Littleton, CO, USA) and rabbit monoclonal anti-β-actin (Sigma-Aldrich, St. Louis, MO, USA) at 4 °C overnight. The membrane was washed with TBS-T buffer 3 times for 5 min, and then was incubated with secondary antibody for 1 h at room temperature. After treatment with enhanced chemiluminescence (ECL) plus Western blot detection reagents (Amersham Bioscience, Freiburg, Germany), the membrane was exposed in LAS-3000 (Fuji Film, Tokyo, Japan).

### 4.5. Evaluation of Dermatitis Severity

The severity of dermatitis was assessed on experimental days 1, 8, 15 and 22. The total clinical severity score for skin lesions was calculated as the sum of the individual scores (graded as 0 (absence), 1 (mild), 2 (moderate), and 3 (severe)) for each of the four AD symptoms (erythema/hemorrhage, scaling/dryness, edema and excoriation/erosion) [18]. Assessment was performed by an investigator who was blinded to the grouping of the experimental animals.

### 4.6. Histological Analysis

Standard procedures were employed for fixation, preparation of tissue sections, deparaffination, hematoxylin/eosin (H&E) staining, toluidine blue staining [16] and immunohistochemical staining [8].

### 4.7. Measurement of Serologic Parameters

Before sacrifice, blood samples were collected after puncturing the heart. Cells were removed from plasma by centrifugation for 10 min at 6000× *g* at 4 °C. The serum levels of immunoglobulin E (IgE) (Bethyl Laboratories Inc., Montgomery, TX, USA) and histamine (Oxford Biomedical research, Inc., Oxford, MI, USA) were measured using specific ELISA kits according to the manufacturer’s instructions. After sacrifice, dorsal skin biopsies were taken from each group. The skin tissues were homogenized and analyzed for the levels of interleukin-17A (IL-17A) using specific ELISA kits (BioLegend, San Diego, CA, USA) according to the manufacturer’s instructions.

### 4.8. Reverse Transcription Polymerase Chain Reaction (RT-PCR)

Total RNA was isolated from dorsal skin using a Trizol^®^ reagent kit (Invitrogen, Carlsbad, CA, USA) according to the manufacturer’s instructions. mRNA concentrations were measured by NanoDrop^®^ ND-2000 UV-Vis Spectrophotometer (Thermo Fisher Scientific, Wilmington, DE, USA). RNA (2.5 μg) was reversibly transcribed with reverse transcriptase and oligo-(dT) primer (Intron Biotechnology, Seoul, Korea). All the primer sequences, product size and references are shown in Table 1. PCR was performed using a PCR premix kit (Intron Biotechnology) for each primer and was terminated by heating to 72 °C for 10 min. PCR products were resolved on a 1.5% agarose gel (USB) and visualized with ultraviolet light after SYBR green-safe^®^ (Invitrogen) staining. 

### 4.9. Statistical Analysis

Data are presented as mean ± standard error of the mean (SEM) of at least three independent experiments. Statistical significance was determined by Tukey’s honestly significant difference (Tukey’s HSD) test using GraphPad Prism 4.0 software (GraphPad Software Inc., version 4.0, San Diego, CA, USA). Data were considered significant if the *p*-value was <0.05.

## Figures and Tables

**Table 1:** The sequences and product size (bp) of primers.

Genes	Sequences of Primers	Product Size (bp)	Reference
IL-4	FW: 5′-AGTTGTCATCCTGCTCTTCTTT-3′ Rev: 5′-GACTGGGACTCATTCATGGTGC-3′	342	[27]
IL-5	FW: 5′-CAAAAAGAGAAGTGTGGCGAGG-3′ Rev: 5′-TAGATAGGAGCAGGAAGCCCG-3′	701	[28]
IL-6	FW: 5′-AGTTGCCTTCTTGGGACTGA-3′ Rev: 5′-TCCACGATTTCCCAGAGAAC-3′	159	[29]
IL-12	FW: 5′-CGGGACCAAACCAGCACATT-3′ Rev: 5′-GCAGAGTCTCGCCATTATGATTCA-3′	307	[30]
IL-13	FW: 5′-GCAACGGCAGCATGGTATGGAG-3′ Rev: 5′-TGGTATAGGGGAGGCTGGAGAC-3′	170	[28]
IFN-γ	FW: 5′-ATCTGGAGGAACTGGCAAAA-3′ Rev: 5′-TTCAAGACTTCAAAGAGTCTGAGGTA-3′	176	[31]
MDC	FW: 5′-TCTGATGCAGGTCCCTATGGT-3′ Rev: 5′-TTATGGAGTAGCTTCTTCAC-3′	206	[32]
TARC	FW: 5′-CAGGAAGTTGGTGAGCTGGTATA-3′ Rev: 5′-TTGTGTTCGCCTGTAGTGCATA-3′	757	[32]
TSLP	FW: 5′-TGCAAGTACTAGTACGGATGGGGC-3′ Rev: 5′-GGACTTCTTGTGCCATTTCCTGAG-3′	324	[33]
β-actin	FW: 5′-GCACCACACCTTCTACAATGAG-3′ Rev: 5′-TTGGCATAGAGGTCTTTACGGA-3′	630	[27]

## Abbreviations

AD	Atopic dermatitis
BALF	Bronchoalveolar lavage fluid
dTCTP	Dimerized translationally controlled tumor protein
dTBP2	dTCTP-binding peptide 2
HRF	Histamine releasing factor
IL	Interleukin
IFN-γ	Interferon-gamma
MDC	Macrophage-derived chemokine
TARC	Thymus and activation-regulated chemokine
TCTP	Translationally controlled tumor protein
TNF-α	Tumor necrosis factor-alpha
TSLP	Thymic stromal lymphopoietin

## References

[1] Barker J.N., Palmer C.N., Zhao Y., Liao H., Hull P.R., Lee S.P., Allen M.H., Meggitt S.J., Reynolds N.J., Trembath R.C. (2007). Null mutations in the filaggrin gene (*FLG*) determine major susceptibility to early-onset atopic dermatitis that persists into adulthood. J. Investig. Dermatol..

[2] Pugliarello S., Cozzi A., Gisondi P., Girolomoni G. (2011). Phenotypes of atopic dermatitis. J. Dtsch. Dermatol. Ges..

[3] Ference J.D., Last A.R. (2009). Choosing topical corticosteroids. Am. Fam. Physician.

[4] Saeki H. (2009). The brief commentary on guidelines for the management of atopic dermatitis 2009—The point of drug treatment. Arerugi.

[5] Pariser D. (2009). Topical corticosteroids and topical calcineurin inhibitors in the treatment of atopic dermatitis: Focus on percutaneous absorption. Am. J. Ther..

[6] Jirachotikoon C., Tannukit S., Kedjarune-Leggat U. (2015). Expression of translationally controlled tumor protein in heat-stressed human dental pulp cells. Arch. Oral Biol..

[7] Chen C., Deng Y., Hua M., Xi Q., Liu R., Yang S., Liu J., Zhong J., Tang M., Lu S. (2015). Expression and clinical role of TCTP in epithelial ovarian cancer. J. Mol. Histol..

[8] Sheverdin V., Jung J., Lee K. (2013). Immunohistochemical localization of translationally controlled tumor protein in the mouse digestive system. J. Anat..

[9] Sheverdin V., Bae S.Y., Shin D.H., Lee K. (2012). Expression and localization of translationally controlled tumor protein in rat urinary organs. Microsc. Res. Tech..

[10] Gnanasekar M., Rao K.V., Chen L., Narayanan R.B., Geetha M., Scott A.L., Ramaswamy K., Kaliraj P. (2002). Molecular characterization of a calcium binding translationally controlled tumor protein homologue from the filarial parasites *Brugiamalayi* and *Wuchereriabancrofti*. Mol. Biochem. Parasitol..

[11] Bommer U.A., Thiele B.J. (2004). The translationally controlled tumour protein (TCTP). Int. J. Biochem. Cell Biol..

[12] Kim M., Min H.J., Won H.Y., Park H., Lee J.C., Park H.W., Chung J., Hwang E.S., Lee K. (2009). Dimerization of translationally controlled tumor protein is essential for its cytokine-like activity. PLoS ONE.

[13] Sampson H.A., Broadbent K.R., Bernhisel-Broadbent J. (1989). Spontaneous release of histamine from basophils and histamine-releasing factor in patients with atopic dermatitis and food hypersensitivity. N. Engl. J. Med..

[14] Kim M., Jin Y.B., Lee K., Lee Y.S. (2013). A new antiallergic agent that binds to dimerized translationally controlled tumor protein and inhibits allergic symptoms is nontoxic. Hum. Exp. Toxicol..

[15] Kim M., Chung J., Lee C., Jung J., Kwon Y., Lee K. (2011). A peptide binding to dimerized translationally controlled tumor protein modulates allergic reactions. J. Mol. Med..

[16] Matsuoka H., Maki N., Yoshida S., Arai M., Wang J., Oikawa Y., Ikeda T., Hirota N., Nakagawa H., Ishii A. (2003). A mouse model of the atopic eczema/dermatitis syndrome by repeated application of a crude extract of house-dust mite *Dermatophagoides farinae*. Allergy.

[17] Suto H., Matsuda H., Mitsuishi K., Hira K., Uchida T., Unno T., Ogawa H., Ra C. (1999). NC/Nga mice: A mouse model for atopic dermatitis. Int. Arch. Allergy Immunol..

[18] Yamamoto M., Haruna T., Yasui K., Takahashi H., Iduhara M., Takaki S., Deguchi M., Arimura A. (2007). A novel atopic dermatitis model induced by topical application with *Dermatophagoides farinae* extract in NC/Nga mice. Allergol. Int..

[19] Kang J.S., Lee K., Han S.B., Ahn J.M., Lee H., Han M.H., Yoon Y.D., Yoon W.K., Park S.K., Kim H.M. (2006). Induction of atopic eczema/dermatitis syndrome-like skin lesions by repeated topical application of a crude extract of *Dermatophagoides pteronyssinus* in NC/Nga mice. Int. Immunopharmacol..

[20] Racke M.K., Bonomo A., Scott D.E., Cannella B., Levine A., Raine C.S., Shevach E.M., Rocken M. (1994). Cytokine-induced immune deviation as a therapy for inflammatory autoimmune disease. J. Exp. Med..

[21] Peng W., Novak N. (2015). Pathogenesis of atopic dermatitis. Clin. Exp. Allergy.

[22] Leung D.Y., Boguniewicz M., Howell M.D., Nomura I., Hamid Q.A. (2004). New insights into atopic dermatitis. J. Clin. Investig..

[23] Kang H.S., Lee M.J., Song H., Han S.H., Kim Y.M., Im J.Y., Choi I. (2001). Molecular identification of IgE-dependent histamine-releasing factor as a B cell growth factor. J. Immunol..

[24] Kashiwakura J.C., Ando T., Matsumoto K., Kimura M., Kitaura J., Matho M.H., Zajonc D.M., Ozeki T., Ra C., MacDonald S.M. (2012). Histamine-releasing factor has a proinflammatory role in mouse models of asthma and allergy. J. Clin. Investig..

[25] Lee T.Y., Kim D.J., Won J.N., Lee I.H., Sung M.H., Poo H. (2014). Oral administration of poly-γ-glutamate ameliorates atopic dermatitis in Nc/Nga mice by suppressing Th2-biased immune response and production of IL-17A. J. Investig. Dermatol..

[26] Nedoszytko B., Sokolowska-Wojdylo M., Ruckemann-Dziurdzinska K., Roszkiewicz J., Nowicki R.J. (2014). Chemokines and cytokines network in the pathogenesis of the inflammatory skin diseases: Atopic dermatitis, psoriasis and skin mastocytosis. Postepy Dermatol. Alergol..

[27] Inoue J., Aramaki Y. (2007). Suppression of skin lesions by transdermal application of CpG-oligodeoxynucleotides in NC/Nga mice, a model of human atopic dermatitis. J. Immunol..

[28] Jeong Y.I., Hong S.H., Cho S.H., Lee W.J., Lee S.E. (2015). *Toxoplasma gondii* infection suppresses house dust mite extract-induced atopic dermatitis in NC/Nga mice. Allergy Asthma Immunol. Res..

[29] Zhong W., Yin H., Xie L. (2009). Expression and potential role of major inflammatory cytokines in experimental keratomycosis. Mol. Vis..

[30] Ait-Oufella H., Wang Y., Herbin O., Bourcier S., Potteaux S., Joffre J., Loyer X., Ponnuswamy P., Esposito B., Dalloz M. (2013). Natural regulatory T cells limit angiotensin II-induced aneurysm formation and rupture in mice. Arterioscler. Thromb. Vasc. Biol..

[31] Annemann M., Wang Z., Plaza-Sirvent C., Glauben R., Schuster M., Sander F.E., Mamareli P., Kuhl A.A., Siegmund B., Lochner M. (2015). IκBNS regulates murine Th17 differentiation during gut inflammation and infection. J. Immunol..

[32] Vestergaard C., Yoneyama H., Murai M., Nakamura K., Tamaki K., Terashima Y., Imai T., Yoshie O., Irimura T., Mizutani H. (1999). Overproduction of Th2-specific chemokines in NC/Nga mice exhibiting atopic dermatitis-like lesions. J. Clin. Investig..

[33] Han N.R., Park J.Y., Jang J.B., Jeong H.J., Kim H.M. (2014). A natural dye, Niram improves atopic dermatitis through down-regulation of TSLP. Environ. Toxicol. Pharmacol..

